# Characteristic of volatile flavor compounds in ‘Fengtangli’ plum (*Prunus salicina* Lindl.) were explored based on GC×GC-TOF MS

**DOI:** 10.3389/fnut.2025.1536954

**Published:** 2025-01-30

**Authors:** Xianfeng Hu, Deyan Li, Yi Ding, Yubo Zhang, Chunguang Ren

**Affiliations:** ^1^College of Agriculture, Anshun University, Anshun, Guizhou, China; ^2^Guizhou Mountain Resources Research Institute, Guiyang, Guizhou, China

**Keywords:** characteristic flavor compounds, ‘Fengtangli’ plum, flavor omics, germplasm resources, ‘Siyueli’ plum

## Abstract

**Introduction:**

The ‘Fengtangli’ plum (*Prunus salicina* Lindl.) is favoured by consumers for its characteristic flavor. The purpose of this study was to explore the characteristics of volatile flavor compounds in ‘Fengtangli’ plum.

**Methods:**

The flavor compounds of both ‘Fengtangli’ and ‘Siyueli’ plums were analyzed using comprehensive two-dimensional gas chromatography time-of-flight mass spectrometry (GC×GC-TOF MS).

**Results:**

The results revealed the presence of 495 volatile flavor compounds in ‘Fengtangli’ plum and 466 in ‘Siyueli’ plum. The relative concentrations of hydrocarbons, alcohols, ketones, and esters in ‘Fengtangli’ plum were significantly elevated compared to those detected in ‘Siyueli’ plum. Moreover, the sensorial attributes of sweetness, citrus, herbal, floral, and fruity notes were more prominent in ‘Fengtangli’ plum relative to those of ‘Siyueli’ plum. Through the integration of differential metabolite analysis and relative odor activity assessment, it is hypothesized that furan-2-pentyl; (*E*)-2-octenal; and 1-octen-3-one may represent the characteristic of volatile flavor compounds in ‘Fengtangli’ plum.

**Discussion:**

The research results may provide a theoretical reference for the development and application of ‘Fengtangli’ plum and the study of the synthesis mechanism of characteristic flavor compounds.

## 1 Introduction

*Prunus salicina* Lindl. is a perennial drupe tree belonging to the *Rosaceae* family, and China ranks first worldwide in both the planting area and plum yield (1–3). Chemical compounds produced by edible plants play an important role in maintaining human health, regulating physiological functions and preventing diseases (4). Plums are abundant in antioxidants, including anthocyanins, flavonoids, and polyphenols, which aid in safeguarding the body against free radicals (5). Additionally, plums are rich in anthocyanins, vitamin C, and various antioxidant compounds that contribute to the prevention of blood stasis and the enhancement of blood circulation (6, 7). The ‘Fengtangli’ plum is favored by consumers for its rich characteristic flavor and crisp meat (8). There are often ‘Siyueli’ on the market pretending to be ‘Fengtangli’ plum, mainly because the appearance of ‘Siyueli’ is similar to ‘Fengtangli’ plum, making it difficult for some consumers to identify. The flavor of fruit is a critical factor in determining overall quality and directly influences consumers’ purchasing decisions (9). Currently, the quality evaluation of ‘Fengtangli’ plum is mostly based on conventional indicators, such as sugar content, titratable acidity, and vitamin C content. Nevertheless, there have been no reports of research related to the characteristic flavor compounds of ‘Fengtangli’ plum.

With the advancement of flavor omics technology, it has become possible to comprehensively analyze the odor characteristics of complex food matrices. Comprehensive two-dimensional gas chromatography time-of-flight mass spectrometry (GC × GC-TOF MS) features high throughput, precision, sensitivity and reproducibility, and is widely used in flavor omics. GC × GC-TOF MS achieves orthogonal separation of compounds by connecting two chromatographic columns with different separation mechanisms independently, which not only improves the separation efficiency but also significantly reduces the peak overlap problem. Moreover, this technique has a high acquisition frequency and fast response speed, enabling high - sensitivity detection within the full mass range, allowing it to identify qualitatively several times more substances than traditional gas chromatography–mass spectrometry (GC–MS). Shen et al. analyzed five kinds of commercially - available grilled mutton skewers by GC × GC-TOF MS and GC - MS. A total of 141 compounds were identified by GC × GC-TOF MS, while only 65 compounds were identified by GC–MS (GC–MS) (10, 11). The technology is extensively utilized in detecting flavors in liquor, fruit, pepper, tea, meat products, and other foods, serving as a crucial technology for analyzing the foundational components of food flavor quality (12–16). Researchers have examined the alterations in flavor, volatile aroma, and microbial community of fermented peppers over varying fermentation durations. The findings indicate that peppers fermented for 2 and 4 years exhibited a greater diversity of volatiles and elevated odor activity, contributing to a more favorable flavor profile (17). Green fermented bean curd was inoculated with the new strain *Weissella confusa* M1 to produce many volatile compounds, of which *n*-hexanal and dimethyl sulphide appeared to be the key flavor compounds of green fermented bean curd (18). In summary, GC × GC-TOF MS technology integrates of comprehensive two-dimensional gas chromatography with the high acquisition frequency of time-of-flight mass spectrometry, providing a powerful tool for the analysis of complex samples.

The objective of this research is to determine the characteristic flavor compounds contained in ‘Fengtangli’ plum. We employed GC × GC-TOF MS technology to analyze samples of ‘Fengtangli’ and ‘Siyueli’ plums, and conducted analyses from aspects such as flavor compound annotation, analysis of the sensory flavor characteristics, screening of differential compounds. The research findings may offer a theoretical basis for further investigation into the development and application of ‘Fengtangli’ plum and the study of the synthesis mechanism of characteristic flavor compounds.

## 2 Materials and methods

### 2.1 Experimental details

Ethanol (99.8%) was acquired from Aladdin Holdings Group Limited, while sodium chloride was obtained from China National Pharmaceutical Group Chemical Reagent Co., Ltd. On June 12th, 2023, the mature fruits of ‘Fengtangli’ and ‘Siyueli’ plum cultivars were harvested from the orchard located in Najian Village, Liuma Town, Zhenning County, Anshun City, Guizhou Province (105.78° E, 25.74° N). A total of 500 g samples were collected for each treatment, with three replicates conducted for each treatment. The samples of ‘Fengtangli’ plum were designated as FTL1, FTL2, and FTL3, while those of ‘Siyueli’ plum were labeled as SYL1, SYL2, and SYL3. Subsequently, both the ‘Fengtangli’ and ‘Siyueli’ plum samples were dispatched to Suzhou Panomik Biomedical Technology Co., Ltd. for analysis.

### 2.2 Morphological observation of ‘Fengtangli’ and ‘Siyueli’ plums

The morphological characteristics of the ‘Fengtangli’ and ‘Siyueli’ plum samples, including fruit shape, apex, suture line, pericarp, and flesh color, were meticulously observed. The distinctions between the two cultivars were comprehensively described, and the experimental findings were visually documented through photography and detailed records.

### 2.3 Preparation of internal standard solution

A precise quantity of deuterated regular hexagon-d13 was dissolved in a 50% aqueous ethanol solution to prepare a single standard mother liquor at a concentration of 10 mg/L, which was subsequently stored at 4°C for future use (19).

### 2.4 Flavor compounds extraction

Samples of ‘Fengtangli’ and ‘Siyueli’ plums were transferred to 15 mL centrifuge tubes and diluted with a saturated sodium chloride solution to achieve an ethanol concentration of 10% (v/v). A 5 mL aliquot of the diluted sample was carefully transferred into a 20 mL headspace sampling bottle, followed by the addition of 10 μL of the internal standard solution. The samples were then incubated at 50°C for 10 min. Prior to sample adsorption, the solid-phase microextraction (SPME) head was conditioned at 270°C for 10 min before being placed in the incubation room. The sample was adsorbed at 50°C for 30 min. Following adsorption, the SPME extraction head was transferred to the gas chromatography (GC) inlet at 250°C and desorbed for 5 min. After injection, the SPME extraction head was aged at 270°C for an additional 10 min.

### 2.5 GC×GC-TOF MS analysis

#### 2.5.1 Chromatographic conditions

The GC × GC-TOF MS chromatographic system comprised a high-resolution time-of-flight (TOF) mass spectrometer, an Agilent 8890A gas chromatograph (Agilent Technologies, Palo Alto, CA, USA), a two-stage jet modulator, and a split-splitless injection module. The separation system was configured with a one-dimensional (1D) column, DB-Heavy Wax (30 m × 250 μm × 0.5 μm; Agilent Technologies, USA), and a two-dimensional (2D) column, RXI-5SILMS (2 m × 150 μm × 0.15 μm; Restek Corporation, USA). High-purity helium was employed as the carrier gas at a flow rate of 1.0 mL/min. The initial temperature of the 1D chromatographic column was set at 40°C and held constant for 3 min. Subsequently, the temperature was ramped up to 250°C at a rate of 5°C per minute and maintained for an additional 5 min. The modulation period was set to 4.0 s, with the temperature consistently maintained at 15°C higher than that of the 2D chromatographic column, while the inlet temperature was established at 250°C (20–22).

#### 2.5.2 Conditions for mass spectrometry

The mass spectrometer detector (LECO, St. Joseph, MI, USA) was operated with both the transmission line and ion source maintained at a temperature of 250°C. The electron bombardment source was set to 70 eV, and the detector voltage was configured to 1984 V. The acquisition rate was 200 spectra per second, and the scanning range of the mass spectrometer extended from 35 to 450 m/z.

### 2.6 Flavor compounds annotation

Flavor compounds are produced through a series of intricate biochemical reactions that transform flavor precursors. These precursors encompass a diverse array of volatile flavor compounds, including hydrocarbons, aldehydes, esters, acids, alcohols, ketones, ethers, phenols, and heterocyclic compounds. The Classyfire software and the PubChem database were utilized to annotate flavor compounds, and the quantity and relative content of these compounds were analyzed (23).

### 2.7 Analysis of the sensory flavor characteristics

Fruit flavor is a sensation experienced after the consumption of fruit, representing a comprehensive sensory impression that encompasses the taste perceived in the mouth, the aroma detected in the nasal cavity, and the sensations mediated by the trigeminal nerve. In this study, a comparative and analytical investigation of the sensory flavors of ‘Fengtangli’ and ‘Siyueli’ plum samples was conducted utilizing the flavor database (FlavorDB). To elucidate the distinctive sensory flavor attributes of flavor compounds, a network diagram that delineates the associations between flavor compounds and sensory characteristics was devised utilizing the igraph package (24).

### 2.8 Screening of differential flavor compounds

Different flavor compounds were screened from the sample substance lists of ‘Fengtangli’ and ‘Siyueli’ plums samples under the conditions of *p* value <0.05 and variable importance in the projection (VIP) > 1 in the t-test or one-way ANOVA tests (25). In this study, each object was classified into one class, and these classes were merged into large objects until the end. The hierarchical clustering diagram of the relative quantitative values of the flavor compounds was obtained by scaling the data set using the pheatmap package.

### 2.9 Data processing

After data analysis with ChromaTOF software, the name of each sample compounds, chemical abstracts service (CAS) number, retention time and database information were obtained, and the peak area of the sample was calculated. The final material information table was created by integrating the annotation information for each sample. The total peak area of data was normalized to compare data of different magnitudes (26).

### 2.10 Statistical analysis

The flavor compounds annotated via flavor omics were obtained from the FlavorDB database.^1^ Principal component analysis (PCA) was employed to classify and downscale the data of flavor compounds to obtain reliable and intuitive results. Variance analysis was conducted using Statistix 8.1 (Tallahassee, FL, USA), with the comparison processing method based on the least significant difference test at a significance level of 0.05.

## 3 Results and discussion

### 3.1 Morphological differences between ‘Fengtangli’ and ‘Siyueli’ plums

**Figure 1:**
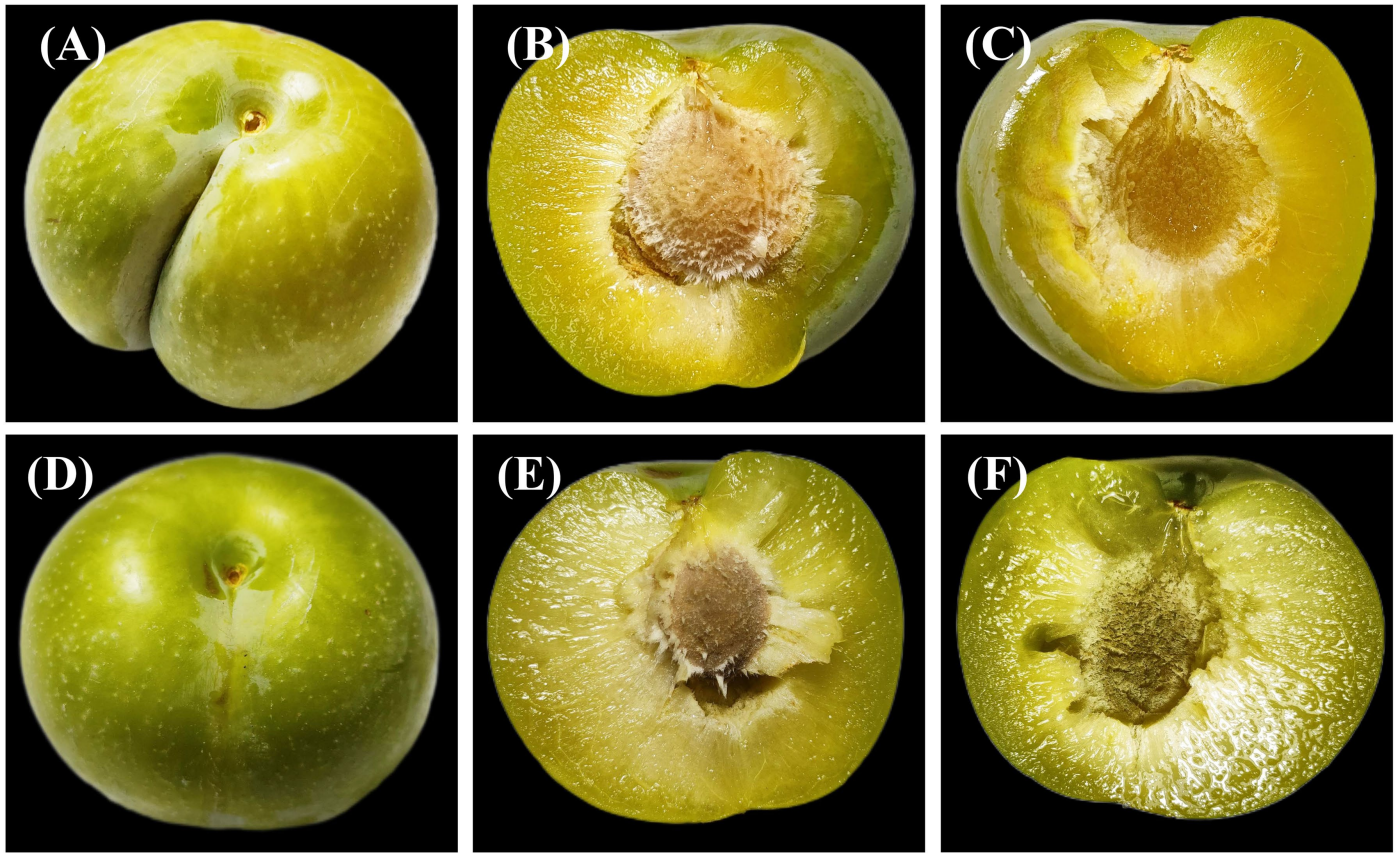
Appearance and morphology of ‘Fengtangli’ and ‘Siyueli’ plums. **(A–C)** ‘Fengtangli’ plum, **(D–F)** ‘Siyueli’ plum.

A significant difference in fruit morphology was observed between ‘Fengtangli’ and ‘Siyueli’ plums. As illustrated in Figure 1, the fruit of ‘Fengtangli’ plum exhibits an oval shape with a flattened apex. The peel displayed a light yellow hue and was adorned with abundant fruit powder. The fruit’s apex was marginally indented, and it featured a pronounced suture that was deep in appearance. Notably, the symmetry of the fruit was prominent, and the separation between the pulp and the core was notably facile. In contrast, the ‘Siyueli’ plum was nearly spherical in shape, featuring a slightly concave apex, a shallow suture, and a smooth yellow - green pericarp that was covered with fruit powder and had a thin texture.

### 3.2 Data inspection and multivariate statistical analysis

**Figure 2:**
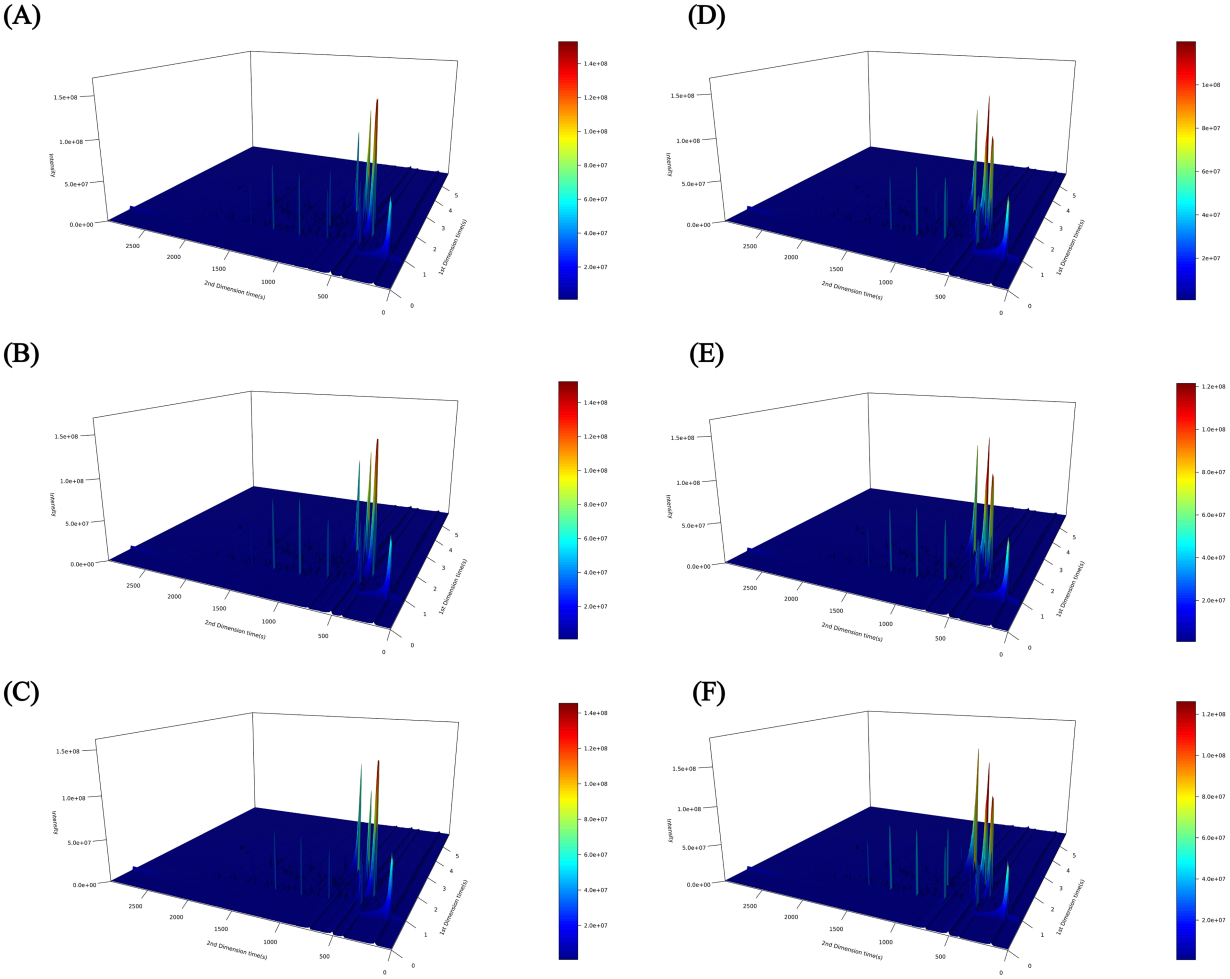
Total ion flow chromatogram of ‘Fengtangli’ and ‘Siyueli’ plums. **(A–C)** ‘Fengtangli’ plum, **(D–F)** ‘Siyueli’ plum.

**Figure 3:**
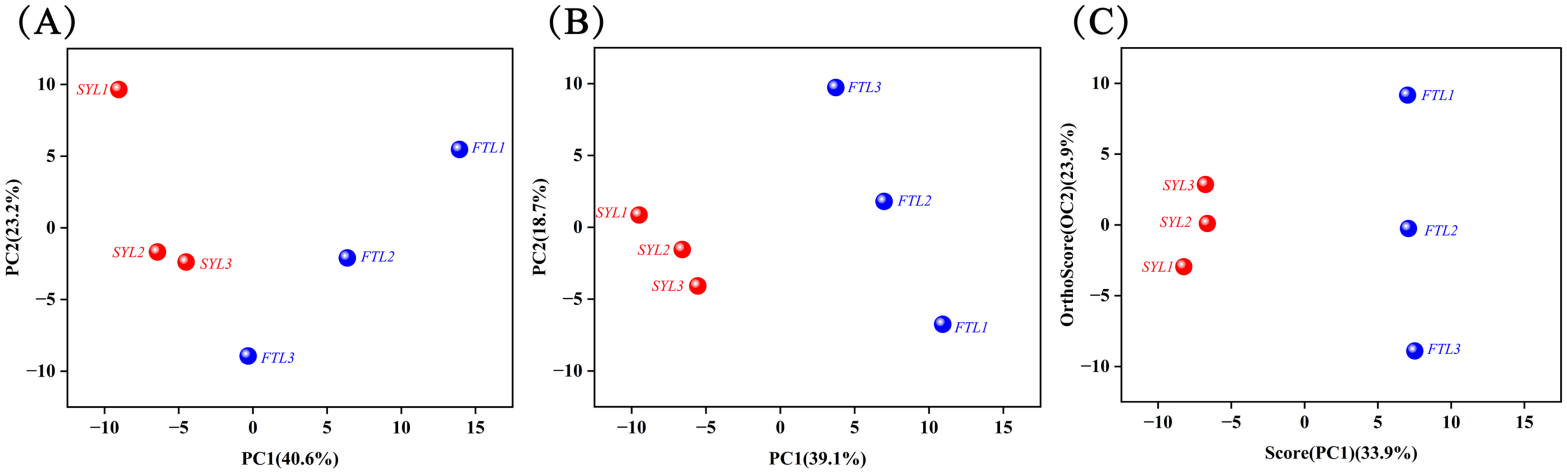
Multivariate statistical analysis chart. **(A)** PCA Score Plot, **(B)** PLS-DA Score Plot, **(C)** OPLS-DA Score Plot. FTL represents ‘Fengtangli’ plum, SYL represents ‘Siyueli’ plum.

The components isolated by chromatography were subsequently introduced into a mass spectrometer, which performed continuous scanning for data collection. A mass spectrogram was generated from each scan, and the ion intensities from all mass spectrograms were aggregated to obtain the total ion current intensity. In this study, GC × GC-TOF MS was employed to analyze the volatile components of ‘Fengtangli’ and ‘Siyueli’ plums. The total ion flow chromatography is shown in Figure 2, the color and peak height in the total ion flow chromatogram indicate the intensity of the ion response. The overall peak appearance frequency in the three - dimensional (3D) total ion flow chromatogram of the samples from ‘Fengtangli’ and ‘Siyueli’ plums was distinctly high, which indicated that a considerable quantity of volatile compounds were contained in both kinds of plums. Principal component analysis (PCA) represents a mathematical technique that is employed for data dimensionality reduction as well as feature extraction (27). This method comprises a linear transformation that maps the original data into a new coordinate system, thereby maximizing the variance of the data in the new space. PCA assists in comprehending the overall characteristics of the data, which is conducive to the identification and elimination of outliers, and thus enhances the accuracy of the model. As an exploratory analysis performed in an unsupervised manner, it is essential to preliminarily assess the differences among various samples prior to model establishment. As illustrated in Figure 3, the PCA score chart serves as a tool for assessing the degree of aggregation and dispersion among the samples. Each point is categorized into two distinct clusters, the ‘Siyueli’ plum samples were positioned on the left side of PC1, while the ‘Fengtangli’ plum samples were situated on the right side of PC1. The PCA results indicate significant differences in flavor compounds between the ‘Siyueli’ and ‘Fengtangli’ plums varieties.

### 3.3 Statistical data of identified compounds of ‘Fengtangli’ and ‘Siyueli’ plums

**Figure 4:**
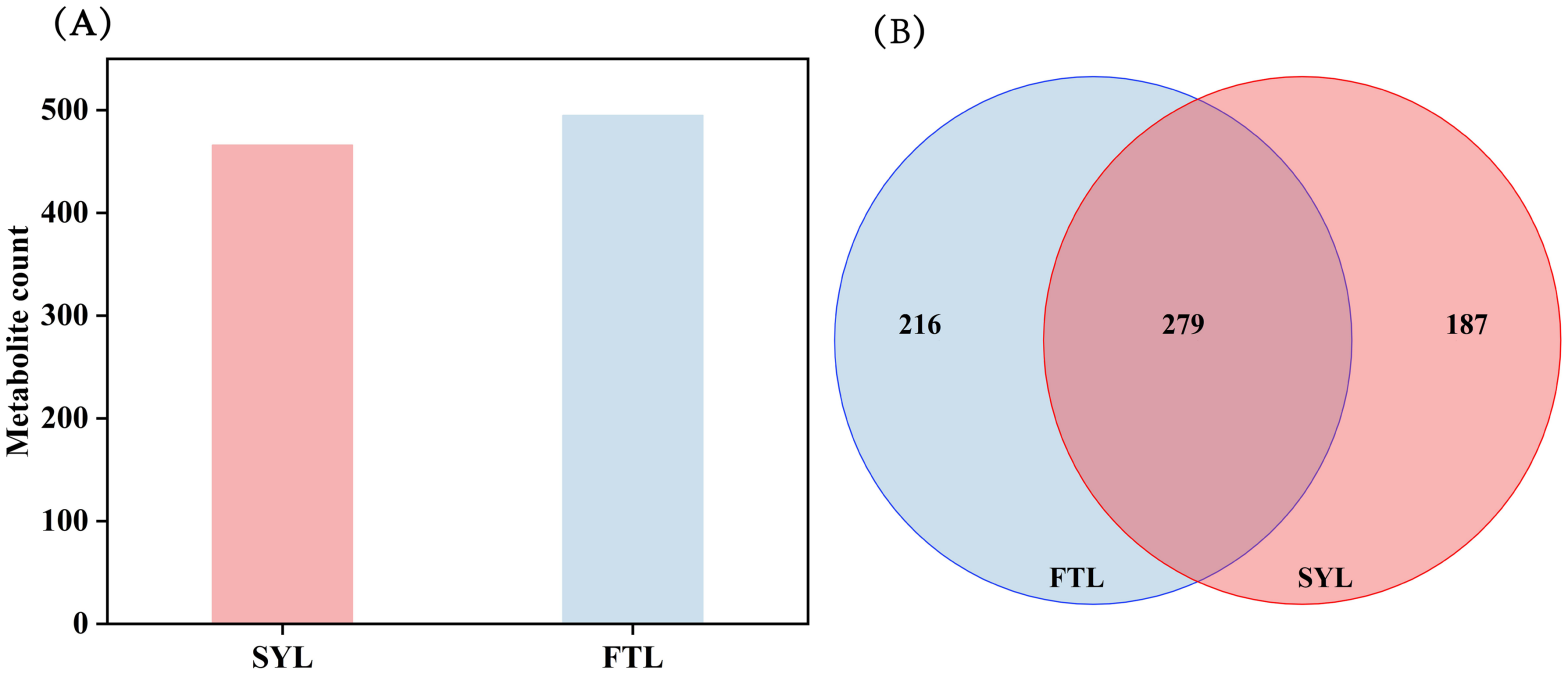
Statistics on the number of identified compounds. **(A)** Statistical chart of identified compounds, and **(B)** venn diagram of identified compounds. FTL represents ‘Fengtangli’ plum, SYL represents ‘Siyueli’ plum.

After the annotation of detected compounds and the removal of impurities, the flavor compounds in ‘Siyueli’ and ‘Fengtangli’ plums were identified. As illustrated in Figure 4, a total of 495 volatile flavor compounds were identified from ‘Fengtangli’ plum, while 466 compounds were detected in ‘Siyueli’ plum. Within the detectable range, the flavor compounds present in ‘Fengtangli’ plum exhibited a greater abundance compared to those in ‘Siyueli’ plum. The Venn diagram illustrates that 279 flavor compounds were identified in both plum cultivars. Additionally, 216 flavor compounds were specific to ‘Fengtangli’ plum, surpassing the 187 specific to ‘Siyueli’ plum. Consequently, the characteristic flavor compounds of ‘Fengtangli’ plum were richer than those of ‘Siyueli’ plum.

### 3.4 Analysis of flavor compounds of ‘Fengtangli’ and ‘Siyueli’ plums

**Figure 5:**
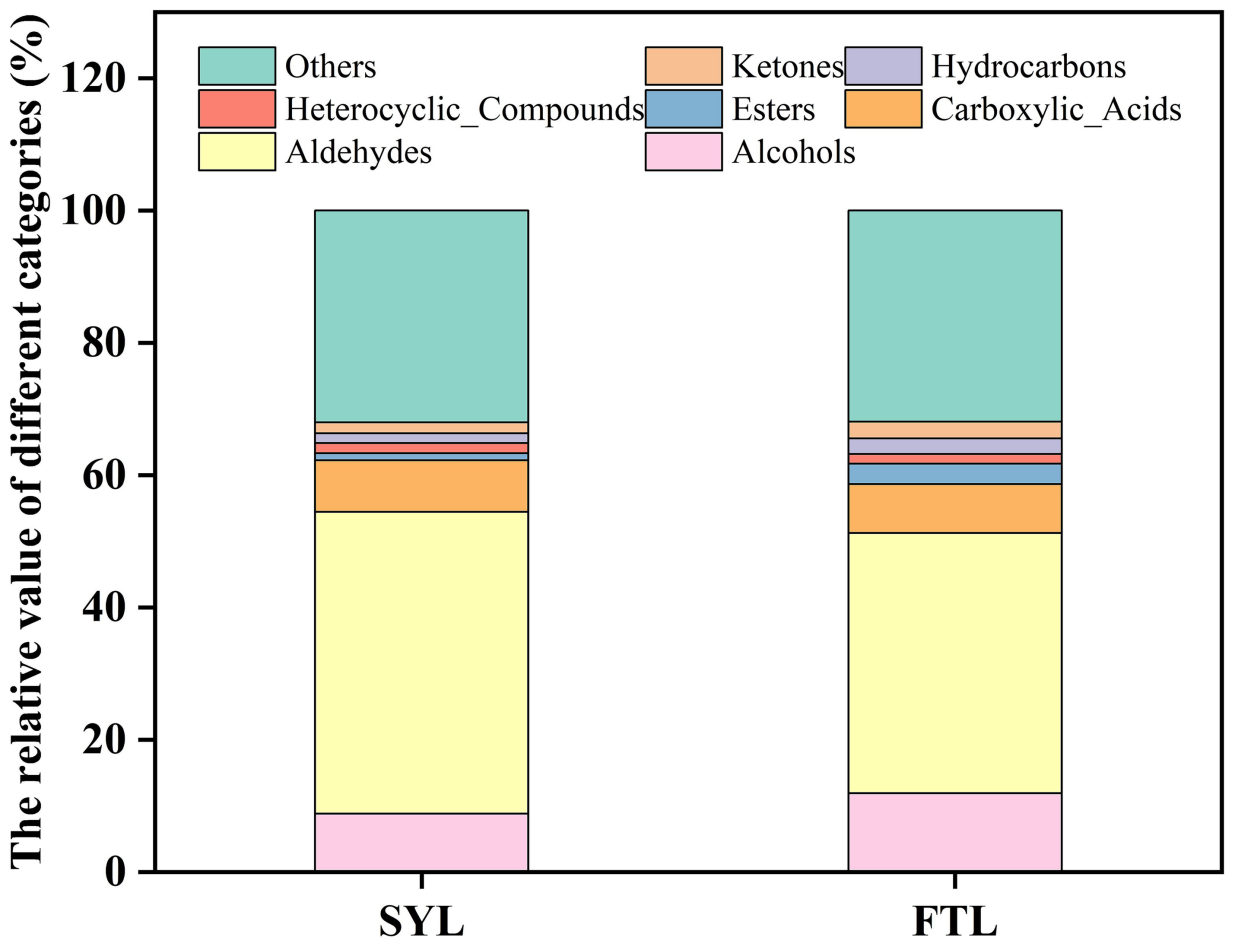
Stacking diagram of relative content of flavor compound types. FTL represents ‘Fengtangli’ plum, SYL represents ‘Siyueli’ plum.

Flavor perception represents a multifaceted sensory process that involves the intricate integration of gustatory cues from the lingual epithelium, olfactory stimuli emanating from the retronasal passage, and somatosensory inputs from the trigeminal nerve system, all of which collectively culminate in a hedonic and discriminative sensory experience (28, 29). Flavor precursors are endogenous or exogenous bioactive molecules that serve as substrates for enzymatic or non-enzymatic chemical transformations, ultimately yielding volatile and non-volatile flavor compounds that contribute to the overall sensory profile of food products (30). The principal flavor constituents present in foodstuffs are synthesized through a complex series of biochemical reactions, which involve the conversion of flavor precursors encompassing a diverse array of chemical classes, including esters, acids, hydrocarbons, ketones, aldehydes, ethers, alcohols, phenols, and heterocyclic compounds (31, 32). The flavor compounds in ‘Fengtangli’ and ‘Siyueli’ plums were analyzed using the PubChem database and Classyfire software to assess the differences in the relative contents of flavor compounds between the two cultivars. As shown in Figure 5, the proportions of hydrocarbons (2.32% *vs*. 1.49%), alcohols (11.90% *vs.* 8.82%), ketones (2.57% *vs.* 1.64%) and esters (3.09% *vs.* 1.07%) in ‘Fengtangli’ plum were higher than those in ‘Siyueli’ plum. Conversely, the relative contents of aldehydes (39.33% *vs.* 45.65%), carboxylic_acids (7.41% *vs.* 7.78%), heterocyclic compounds (1.48% *vs.* 1.54%), and other volatile compounds (31.90% *vs.* 32.02%) in ‘Fengtangli’ plum were lower than those in ‘Siyueli’ plum. The flavor compounds of fruits are mainly esters, including fatty acids and aromatic esters (33). Esters in fruits are mainly produced from unsaturated fatty acids through the lipoxygenase (LOX) and *β*-oxidation pathways (34). Esters can also be formed through the esterification reaction between acids and alcohols. Lipoxygenase (LOX) catalyzes the conversion of fatty acids into aldehydes, which are subsequently reduced to alcohols by alcohol dehydrogenase (ADH). In the presence of acyl-CoA (Coenzyme A), these alcohols are further converted into esters under the catalysis of alcohol acyltransferase (AAT) (35). Alcohols serve as vital aromatic constituents within fruits, endowing them with their delicate and enticing fragrances (36). It can be inferred that compounds such as esters, alcohols, and ketones likely contribute positively to the characteristic flavor of ‘Fengtangli’ plum.

### 3.5 Screening of differential compounds between ‘Fengtangli’ and ‘Siyueli’ plums

**Figure 6:**
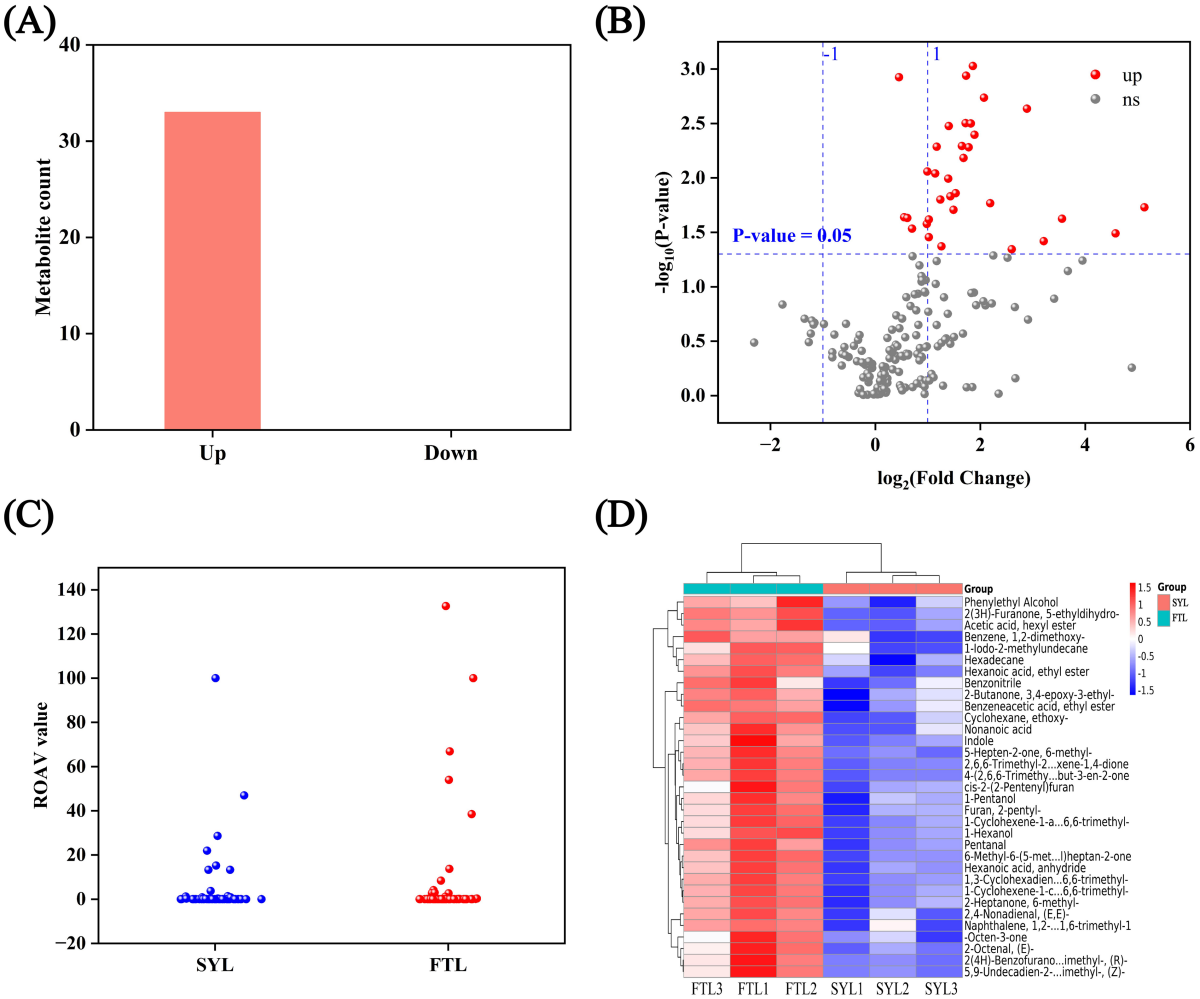
Screening of differential compounds between ‘Fengtangli’ and ‘Siyueli’ plums. **(A)** Statistical chart of the number of differential flavor compounds. **(B)** Volcano plot of flavor compounds. **(C)** Volcano plot of volatile threshold analysis. **(D)** Heat map of differential flavor compounds. FTL represents ‘Fengtangli’ plum, SYL represents ‘Siyueli’ plum.

Through screening, the differential compounds were found from the sample of ‘Fengtangli’ and ‘Siyueli’ plum. As shown in Figure 6, the ‘Fengtangli’ plum was compared with the ‘Siyueli’ plum, 33 flavor compounds were up-regulated and 0 flavor compounds were down-regulated. The 33 up-regulated flavor compounds in ‘Fengtangli’ plum are listed in Table 1. The volcano diagram of differential flavor compounds shows that the rows correspond to flavor compounds while the columns represent sample information. The color gradient indicates the abundance of these flavor compounds, with darker colors signifying higher abundance and lighter colors indicating lower abundance. Additionally, similar flavor compounds or samples may cluster together, suggesting that they share comparable expression patterns (Figure 6D). As illustrated in Figure 6C and Supplementary Table S1, the screening of aroma characteristic compounds with odor activity values (OAVs) ≥ 30 revealed that furan-2-pentyl, heptanal, (*E*)-2-octenal, and 1-octen-3-one significantly contribute to the flavor profile of ‘Fengtangli’ plum. Combining differential metabolite and relative odor activity analysis, it is speculated that furan-2-pentyl, (*E*)-2-octenal, 1-octen-3-one may be the characteristic flavor compounds that distinguish ‘Fengtangli’ plum from ‘Siyueli’ plum. The furan-2-pentyl was produced in the baking process of sweet potato, which made the sweet potato taste sweeter and the aroma richer (37–39). Aroma reorganization and omission experiments showed that (*E*)-2-octenal was the key aroma components of the *Amomum tsao-ko* samples (40). The effects of various cooking methods on the aroma components of *Agaricus bisporus* were investigated, resulting in the detection of 73 distinct aroma components. The analysis of OAVs indicated that 1-octen-3-one is likely the primary contributor to the aroma of roasted *Agaricus bisporus* (41).

**Table 1:** Statistical analysis of different flavor compounds.

Flavor compounds name	Class
2(4*H*)-benzofuranone, 5,6,7,7a-tetrahydro-4,4,7a-trimethyl-, (*R*)-	Heterocyclic_Compounds
Naphthalene, 1,2-dihydro-1,1,6-trimethyl-	Benzenoids
Benzene, 1,2-dimethoxy-	Benzenoids
1-iodo-2-methylundecane	Organohalogen compounds
1-hexanol	Alcohols
1-pentanol	Alcohols
1-octen-3-one	Ketones
1-cyclohexene-1-acetaldehyde, 2,6,6-trimethyl-	Aldehydes
2,6,6-trimethyl-2-cyclohexene-1,4-dione	Ketones
Furan, 2-pentyl-	Heterocyclic_Compounds
2-octenal, (*E*)-	Aldehydes
2-butanone, 3,4-epoxy-3-ethyl-	Ketones
4-(2,6,6-trimethylcyclohexa-1,3-dienyl)but-3-en-2-one	Lipids and lipid-like molecules
5-hepten-2-one, 6-methyl-	Ketones
6-methyl-6-(5-methylfuran-2-yl)heptan-2-one	Heterocyclic_Compounds
2-heptanone, 6-methyl-	Ketones
1-Cyclohexene-1-carboxaldehyde, 2,6,6-trimethyl-	Organic oxygen compounds
2(3*H*)-Furanone, 5-ethyldihydro-	Heterocyclic_Compounds
Benzonitrile	Benzenoids
Phenylethyl Alcohol	
Benzeneacetic acid, ethyl ester	Benzenoids
2,4-Nonadienal, (*E*,*E*)-	Aldehydes
Hexanoic acid, anhydride	Carboxylic_Acids
Hexanoic acid, ethyl ester	Esters
Nonanoic acid	Lipids and lipid-like molecules
5,9-Undecadien-2-one, 6,10-dimethyl-, (*Z*)-	Lipids and lipid-like molecules
Hexadecane	Hydrocarbons
Cis-2-(2-Pentenyl)furan	
Pentanal	Aldehydes
Acetic acid, hexyl ester	Esters
Cyclohexane, ethoxy-	Ethers
Indole	Heterocyclic_Compounds
1,3-Cyclohexadiene-1-carboxaldehyde, 2,6,6-trimethyl-	Organic oxygen compounds

### 3.6 Analysis of the sensory flavor of ‘Fengtangli’ and ‘Siyueli’ plums

**Figure 7:**
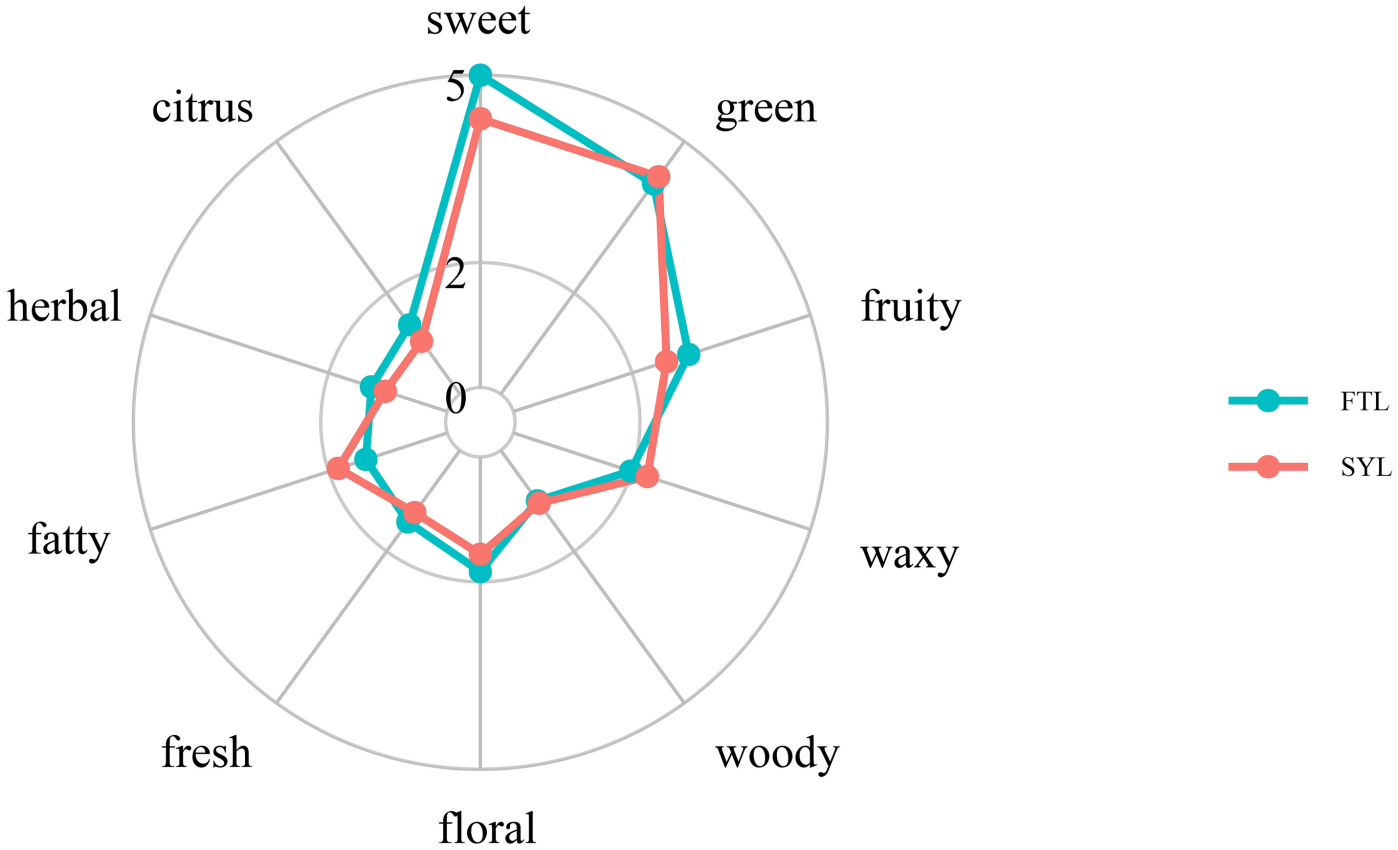
The radar chart of sensory flavor characteristic analysis. FTL represents ‘Fengtangli’ plum, SYL represents ‘Siyueli’ plum.

**Figure 8:**
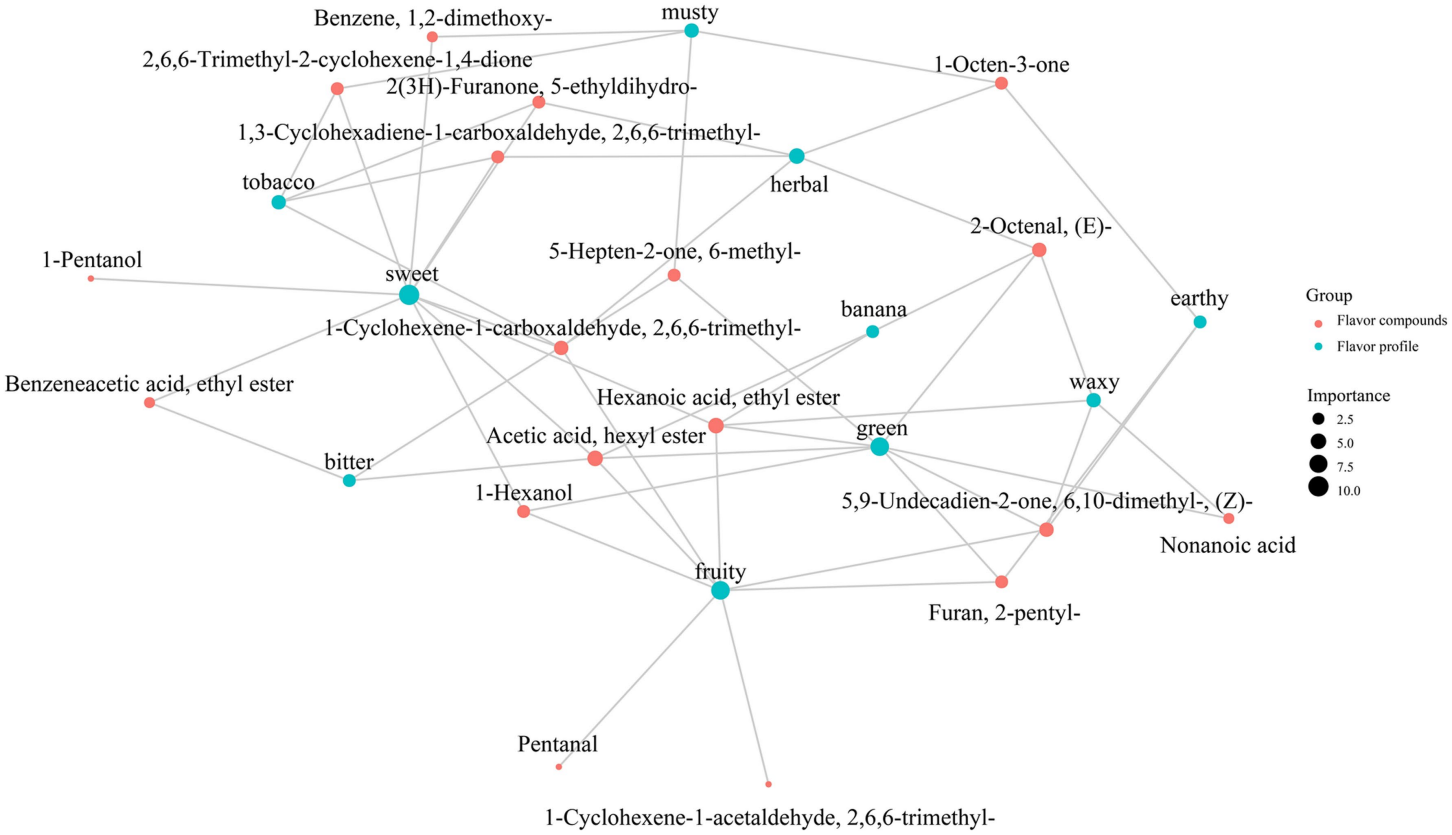
Correlation network diagram between sensory and flavor compounds.

The volatile flavor compounds in fruits, including esters, alcohols, carbonyl compounds, acids and terpenes, are sensed through olfacory receptors on the nasal mucosa. Aroma compounds are usually described as floral, sweet, green, fruity and fatty according to aroma types. The sensory flavor of compounds in ‘Fengtangli’ and ‘Siyueli’ plums were compared against the database of flavor molecules in FlavorDB to clarify the differences in sensory flavor characteristics. Ten attributes, specifically sweet, fruity, green, waxy, fatty, woody, fresh, citrus, herbal and floral, were employed to characterize the sensory flavor profile of ‘Fengtangli’ and ‘Siyueli’ plums. Compared to ‘Siyueli’ plum, ‘Fengtangli’ plum displayed enhanced sweet, citrus, herbal, floral, and fruity notes, while showing reduced waxy and fatty flavors, and maintaining similar woody and green characteristics (Figure 7). The network diagram illustrating the relationship between flavor compounds and sensory characteristics was constructed using the igraph network analysis software. As illustrated in Figure 8, red circles denote flavor compounds, with larger circles indicating a higher number of associated sensory characteristics. Green circles represent sensory attributes, where the size of the green circle is directly proportional to the diversity of flavor compounds connected to that particular sensory attribute. In ‘Fengtangli’ plum, (*E*)-2-octenal was associated with banana, green, herbal, waxy and other flavor characteristics; 1-octen-3-one was correlated with herbal, earthy and other flavor attributes; furan-2-pentyl was linked with fruity, green, waxy, earthy and other flavor features.

## 4 Conclusion

The ‘Fengtangli’ plum is favored by consumers for its sweet, fruity, and rich flavor. Nevertheless, research pertaining to the distinctive flavor compounds of ‘Fengtangli’ plum remains unclear. In this study, the differentiation between the ‘Fengtangli’ and the ‘Siyueli’ plums was carried out by employing GC × GC-TOF MS. A total of 279 kinds of flavor compounds were identified across both plum cultivars. Moreover, a total of 216 distinct flavor compounds were exclusively identified in ‘Fengtangli’ plum, which outnumbers the 187 unique compounds found in ‘Siyueli’ plums. The relative contents of hydrocarbons (2.32% *vs.* 1.49%), alcohols (11.90% *vs.* 8.82%), ketones (2.57% *vs.* 1.64%) and esters (3.09% *vs.* 1.07%) in the ‘Fengtangli’ plum were higher than those in the ‘Siyueli’ plum. In contrast to ‘Siyueli’ plum, ‘Fengtangli’ plum displayed a more pronounced bouquet of sensory flavors, such as sweetness, citrus, herbal, floral, and freshness, while showing less distinct flavors of wax and fat. The woody and green flavors of the ‘Fengtangli’ plum were comparable to those of the ‘Siyueli’ plum. Through the integration of differential metabolite analysis and relative odor activity assessment, it is hypothesized that furan-2-pentyl, (*E*)-2-octenal, and 1-octen-3-one may represent the characteristic of volatile flavor compounds in ‘Fengtangli’ plum. Furthermore, these insights are expected to play a beneficial role in the development and application of ‘Fengtangli’ plum and the study of the synthesis mechanism of characteristic flavor compounds.

## Data Availability

The original contributions presented in the study are included in the article/Supplementary material, further inquiries can be directed to the corresponding authors.

## Footnotes

^1^ https://cosylab.iiitd.edu.in/flavordb/
