# Supplementary material for: Characteristic of volatile flavor compounds in ‘Fengtangli’ plum (Prunus salicina Lindl.) were explored based on GC×GC-TOF MS
Source: Front Nutr. 2025 Jan 30;12:1536954. doi: 10.3389/fnut.2025.1536954 (PMC11821493; doi:10.3389/fnut.2025.1536954)
Supplement: Supplementary file 1 [file Table_1.DOC]

Supplementary Material

# Supplementary Tables

| **Table S1.** Threshold analysis of volatiles | | | | |
| --- | --- | --- | --- | --- |
| Name | Class | CAS | SYL_ROAV | FTL_ROAV |
| Phthalic anhydride |  | 85-44-9 | 0.0000341 | 0.000052 |
| Benzene, 1,2-dichloro- | Benzenoids | 95-50-1 | 0.000158 | 0.000030 |
| Benzene, 1,4-dichloro- | Benzenoids | 106-46-7 | 0.000792 | 0.002526 |
| 1-Heptanol | Alcohols | 111-70-6 | 0.004945 | 0.002314 |
| 1-Hexanol | Alcohols | 111-27-3 | 1.210416 | 2.642948 |
| 1-Pentanol | Alcohols | 71-41-0 | 0.022177 | 0.045591 |
| 1-Octanol | Alcohols | 111-87-5 | 0.048788 | 0.116980 |
| 1-Octen-3-ol | Alcohols | 3391-86-4 | 0.016634 | 0.024265 |
| 1-Octen-3-one | Ketones | 4312-99-6 | 15.218685 | 38.473965 |
| 2,6-Nonadienal, (*E*,*Z*)- | Aldehydes | 557-48-2 | 1.358659 | 1.101175 |
| 2-Butoxyethyl acetate | Esters | 112-07-2 | 8.556569 | 5.632604 |
| 2-Heptanone | Ketones | 110-43-0 | 0.023675 | 0.089173 |
| 2-Hexanone | Ketones | 591-78-6 | 0.001389 | 0.000755 |
| 2-Hexenal, (*E*)- | Aldehydes | 6728-26-3 | 0.006155 | 0.010057 |
| Naphthalene, 2-methyl- | Benzenoids | 91-57-6 | 0.009459 | 0.009122 |
| 2-Nonenal, (*E*)- | Aldehydes | 18829-56-6 | 100 | 100 |
| 2-Undecanone | Ketones | 112-12-9 | 0.304288 | 0.138390 |
| Furan, 2-pentyl- | Heterocyclic_Compounds | 3777-69-3 | 28.622096 | 132.655035 |
| 2-Pentanone | Ketones | 107-87-9 | 0.000948 | 0.000022 |
| 2-Octenal, (*E*)- | Aldehydes | 2548-87-0 | 21.934417 | 53.964214 |
| 1-Hexanol, 2-ethyl- | Alcohols | 104-76-7 | 13.290277 | 13.718024 |
| Citral | Lipids and lipid-like molecules | 5392-40-5 | 0.065677 | 0.025112 |
| Terpinen-4-ol | Lipids and lipid-like molecules | 562-74-3 | 3.133224 | 2.410755 |
| 5-Hepten-2-one, 6-methyl- | Ketones | 110-93-0 | 0.002711 | 0.007209 |
| a-Methylstyrene | Benzenoids | 98-83-9 | 0.000127 | 0.000071 |
| a-Terpineol | Lipids and lipid-like molecules | 98-55-5 | 0.004155 | 0.000603 |
| 3-Buten-2-one, 4-(2,6,6-trimethyl-1-cyclohexen-1-yl)- | Lipids and lipid-like molecules | 14901-07-6 | 0.020436 | 0.000072 |
| 2(3*H*)-Furanone, 5-butyldihydro- | Heterocyclic_Compounds | 104-50-7 | 0.000173 | 0.000086 |
| 2(3*H*)-Furanone, 5-ethyldihydro- | Heterocyclic_Compounds | 695-06-7 | 8.556569 | 3.535729 |
| 2(3*H*)-Furanone, dihydro-5-pentyl- | Heterocyclic_Compounds | 104-61-0 | 0.000112 | 0.000029 |
| Benzothiazole | Heterocyclic_Compounds | 95-16-9 | 5.535365 | 0.000010 |
| Phenol | Benzenoids | 108-95-2 | 0.004582 | 0.008634 |
| Benzaldehyde | Benzenoids | 100-52-7 | 13.273555 | 8.371009 |
| Benzoic acid, ethyl ester | Esters | 93-89-0 | 7.217463 | 0.000014 |
| Benzeneacetaldehyde | Aldehydes | 122-78-1 | 3.717887 | 4.086700 |
| Benzeneacetic acid, ethyl ester | Benzenoids | 101-97-3 | 0.0000701 | 0.000195 |
| Acetophenone | Ketones | 98-86-2 | 0.168484 | 0.282366 |
| Styrene | Benzenoids | 100-42-5 | 0.019482 | 0.000215 |
| 1,2-Propanediol, dinitrate | Organic oxygen compounds | 6423-43-4 | 8.603323 | 0.003906 |
| Butanoic acid, hexyl ester | Esters | 2639-63-6 | 2.355434 | 0.000010 |
| Eugenol | Benzenoids | 97-53-0 | 0.000083 | 0.000085 |
| Linalool | Lipids and lipid-like molecules | 78-70-6 | 0.002583 | 0.002431 |
| Heptanal | Aldehydes | 111-71-7 | 46.949598 | 66.870394 |
| Heptanoic acid, ethyl ester | Esters | 106-30-9 | 1.708992 | 5.420721 |
| Decanoic acid, ethyl ester | Esters | 110-38-3 | 1.590522 | 1.434973 |
| Cyclohexanol | Alcohols | 108-93-0 | 0.021006 | 0.000010 |
| Cyclohexanone | Ketones | 108-94-1 | 0.000969 | 0.001744 |
| Hexanal | Aldehydes | 66-25-1 | 0.831332 | 0.943965 |
| Hexanoic acid | Lipids and lipid-like molecules | 142-62-1 | 6.182488 | 0.000025 |
| Hexanoic acid, butyl ester | Esters | 626-82-4 | 2.994667 | 5.556385 |
| Hexanoic acid, hexyl ester | Esters | 6378-65-0 | 1.074277 | 3.409820 |
| Hexanoic acid, ethyl ester | Esters | 123-66-0 | 0.005187 | 0.007526 |
| Toluene | Benzenoids | 108-88-3 | 0.005926 | 0.007194 |
| Hydrazine, methyl- | Organonitrogen compounds | 60-34-4 | 0.000058 | 0.000674 |
| Benzene, (1-methylethyl)- | Benzenoids | 98-82-8 | 0.000376 | 0.000247 |
| Biphenyl | Benzenoids | 92-52-4 | 0.003217 | 0.004349 |
| Dibutyl phthalate | Esters | 84-74-2 | 0.021050 | 0.021947 |
| Diethyl Phthalate | Esters | 84-66-2 | 6.562236 | 0.000017 |
| Naphthalene | Benzenoids | 91-20-3 | 0.027484 | 0.020402 |
| Limonene | Lipids and lipid-like molecules | 138-86-3 | 0.000113 | 0.001778 |
| Nonanal | Aldehydes | 124-19-6 | 0.238662 | 0.218646 |
| Nonanoic acid, ethyl ester | Esters | 123-29-5 | 5.852747 | 3.723636 |
| Nonane | Hydrocarbons | 111-84-2 | 9.429319 | 0.000013 |
| 2-Propenoic acid, 3-phenyl-, ethyl ester | Esters | 103-36-6 | 0.000185 | 0.012121 |
| Methyl salicylate | Esters | 119-36-8 | 0.000089 | 0.000107 |
| Pentanal | Aldehydes | 110-62-3 | 0.822619 | 2.781359 |
| Octanal | Aldehydes | 124-13-0 | 0.116619 | 0.130665 |
| Octanoic acid | Lipids and lipid-like molecules | 124-07-2 | 4.060768 | 4.684313 |
| Octane | Hydrocarbons | 111-65-9 | 0.000345 | 0.000566 |
| Acetic anhydride | Carboxylic_Acids | 108-24-7 | 0.000012 | 0.000012 |
| Acetic acid, hexyl ester | Esters | 142-92-7 | 0.000017 | 0.000163 |
| Acetic acid, pentyl ester | Esters | 628-63-7 | 0.000029 | 0.004932 |
| Isobutyl acetate | Esters | 110-19-0 | 0.000025 | 0.000594 |
| Hexadecanoic acid, ethyl ester | Esters | 628-97-7 | 0.002934 | 0.002701 |
| 2-Buten-1-one, 1-(2,6,6-trimethyl-1,3-cyclohexadien-1-yl)- | Ketones | 23696-85-7 | 0.173751 | 3.004295 |
| Octanoic acid, ethyl ester | Heterocyclic_Compounds | 106-32-1 | 0.000040 | 0.000076 |
